# A journal to highlight health research in EAC countries

**DOI:** 10.24248/EAHRJ-D-17-00074

**Published:** 2017-03-01

**Authors:** Gibson S. Kibiki

**Affiliations:** a Executive Secretary, East African Health Research Commission, East African Community

We are pleased to present The *East African Health Research Journal (EAHRJ)*, a new no-fee, open-access, peer-reviewed journal that focuses on research conducted in and relevant to East Africa. Established by the East African Health Research Commission (EAHRC) of the East African Community (EAC; www.eac.int), the primary aim of the journal is to present evidence that can be the basis for better health policy and practice in the countries of the EAC. The journal also aims to:

- represent the East African perspective on health and health related-issues,
- provide information that is relevant to the EAC,
- be a platform for sharing and dissemination of knowledge,
- enable scholarly recognition of professionals and institutions,
- support professionals’ career development,
- provide forum for health professionals from East Africa to be more visible globally, and
- provide direction in setting up health priorities in the region.

The *EAHRJ* will promote and facilitate the application of knowledge from research to strengthen national and regional health policy and practice; development of human-resource capacities and skills; exchange and dissemination of health-research information; and advocacy of evidence generated from health research.

Issues of the journal will include original articles, reviews, short communications, surveys, commentaries, opinions, book reviews, essays, special and supplementary issues, and reports, and cover a broad range of health and related aspects, including medicine, geo-medicine, dentistry, nursing, pharmacology, toxicology, pharmaceutical science, veterinary science, food science, environment, health-related agriculture science, and public health.

The *EAHRJ* will be published three times a year online, at www.eahealth.org, and in hard copy. Hard copies will be distributed to all relevant stakeholders, such as government institutions and organisations, research institutions, academic institutions, relevant NGOs, civil society organisations, etc.

We are publishing the first issue of the journal in conjunction with the 6th East African Health and Scientific Conference (EAHSC) taking place in Bujumbura, The Republic of Burundi, 29–31 March 2017. The theme of the conference is Preparedness for, and control of disease outbreaks, epidemics, and pandemics in the context of climate change, globalisation, and gaps in health systems. We have aligned the articles published in the first issue of the EAHRJ with the theme of the conference.

We expect that this journal will add value to the various initiatives taken to improve health and wellbeing of the people of East Africa and the world in general.

